# Multivalent interactions of the SUMO-interaction motifs in RING finger protein 4 determine the specificity for chains of the SUMO

**DOI:** 10.1042/BJ20130753

**Published:** 2013-12-10

**Authors:** Kirstin Keusekotten, Veronika N. Bade, Katrin Meyer-Teschendorf, Annie Miriam Sriramachandran, Katrin Fischer-Schrader, Anke Krause, Christiane Horst, Günter Schwarz, Kay Hofmann, R. Jürgen Dohmen, Gerrit J. K. Praefcke

**Affiliations:** *Center for Molecular Medicine Cologne (CMMC), University of Cologne, 50674 Köln, Germany; †Institute for Genetics, University of Cologne, 50674 Köln, Germany; ‡Institute for Biochemistry, University of Cologne, 50674 Köln, Germany

**Keywords:** avidity, promyelocytic leukaemia, protein degradation, RING finger protein 4 (RNF4), small ubiquitin-like modifier (SUMO), SUMO-targeted ubiquitin ligase, APL, acute promyelocytic leukaemia, HA, haemagglutinin, ITC, isothermal titration calorimetry, NB, nuclear body, PIAS, protein inhibitor of activated STAT (signal transducer and activator of transcription), PML, promyelocytic leukaemia, RARα, retinoic acid receptor α, RNF4, RING finger protein 4, SEC, size-exclusion chromatography, SIM, SUMO-interaction motif, STUbL, SUMO-target ubiquitin ligase, SUMO, small ubiquitin-related modifier, wt, wild-type

## Abstract

RNF4 (RING finger protein 4) is a STUbL [SUMO (small ubiquitin-related modifier)-targeted ubiquitin ligase] controlling PML (promyelocytic leukaemia) nuclear bodies, DNA double strand break repair and other nuclear functions. In the present paper, we describe that the sequence and spacing of the SIMs (SUMO-interaction motifs) in RNF4 regulate the avidity-driven recognition of substrate proteins carrying SUMO chains of variable length.

## Abstract 2

The ubiquitin ligase RNF4 targets proteins for proteasomal degradation if they are modified with SUMO chains. RNF4 recognizes its substrates by using short peptide motifs that interact non-covalently with SUMO chains if they contain at least two SUMO moieties.

## INTRODUCTION

Protein modifications by ubiquitin and Ubl (ubiquitin-like) proteins are involved in the regulation of most cellular pathways including cell cycle progression, transcription, DNA repair and stress responses, and their misregulation is implicated in cancer and aging [1–4]. Both ubiquitin and SUMO (small ubiquitin-related modifier) can form substrate-attached chains, in which one unit of the respective modifier is attached to a lysine residue of another. For ubiquitin, the most abundant ubiquitin chain is linked via Lys^48^ which targets its substrate proteins to the proteasome. Mono-ubiquitylation as well as poly-ubiquitylation formed via other lysine residues or linear chains via the N-terminus of ubiquitin confer different regulatory functions [5,6].

SUMO chains are formed mainly via Lys^11^ of the nearly identical isoforms SUMO2 and SUMO3 (referred to as SUMO2/3 from hereon), which is part of a SUMOylation consensus site. A similar attachment site is not present in SUMO1. As a result, SUMO1 does not form chains efficiently [7]. However, SUMO1 can be attached to lysine residues within SUMO2/3 chains leading to chain termination [8]. SUMO is recognized by short SIMs (SUMO-interaction motifs) that have specific consensus sequences [9] and bind with medium to low affinity with dissociation constants in the range 1–100 μM [10–16]. Upon binding to a hydrophobic groove present in SUMO1 as well as SUMO2/3, the unstructured SIMs adopt a β-strand conformation that forms a parallel (SIMa and SIMb) or antiparallel (SIMr) β-sheet together with the β2-strand of SUMO.

The concept that SUMO and ubiquitin systems work independently of one another (or, under certain conditions, antagonistically) changed with the discovery of STUbLs (SUMO-target ubiquitin ligases) [17–20]. These proteins combine RING finger domains allowing ubiquitin ligase activity and SIMs to recognize their SUMOylated substrates. STUbLs are conserved in all eukaryotes, and the mammalian STUbL RNF4 (RING finger protein 4) complements deletions of STUbL genes in yeast [17,19,20]. RNF4 localizes to PML (promyelocytic leukaemia)-NBs (nuclear bodies) [21], and we and others have identified the PML protein and the oncogenic PML-RARα (retinoic acid receptor α) fusion protein as its substrates [22–25]. The SUMO-dependent ubiquitylation of PML by RNF4 can be stimulated by treatment with arsenic trioxide which is also used for the therapy of APL (acute PML) patients [26,27].

**Figure 1:**
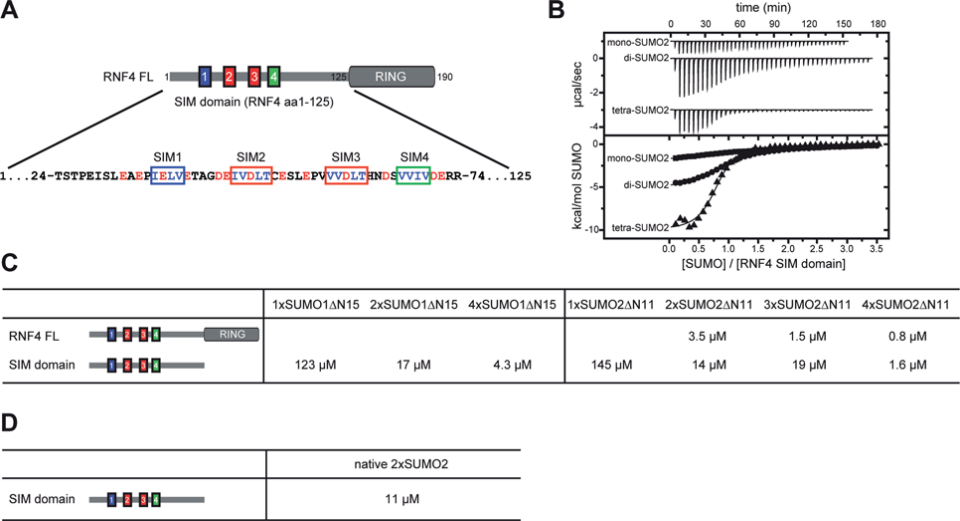
Calorimetric titration of RNF4 with poly-SUMO chains (**A**) Domain architecture of human RNF4. The different types of SIMs are indicated as green (SIMa), red (SIMb) and blue (SIMr) boxes (top panel). Primary sequence of the SUMO-binding region of RNF4 (bottom panel). The core residues are indicated in blue, except for acidic residues which are in red. (**B**) Profiles of typical calorimetric titrations of mono-SUMO2, di-SUMO2 and tetra-SUMO2 into a solution of RNF4 1–125 (upper panel, top to bottom) and the integrated data, normalized to the concentration of the SUMOs (lower panel). (**C** and **D**) Dissociation constants of the interaction of polymeric chains of SUMO1 and SUMO2 with full-length RNF4 (FL) and the SIM domain.

Recent studies showed that RNF4 is also recruited to sites of DNA repair where it regulates ubiquitylation and, at least in some cases, the turnover of repair factors [28–32]. A proteomic analysis of poly-SUMOylated proteins identified several hundred additional putative substrates for RNF4 and other STUbLs. Especially after heat stress these poly-SUMOylated proteins were detected as high-molecular mass SUMO2/3 conjugates [33]. The C-terminal RING finger in RNF4 promotes homo-dimerization and the interaction with E2 enzymes [34–37]. The N-terminal part of RNF4 contains four putative SIMs (see Figure 1A). However, not all SIMs contribute equally to binding [24,38].

In the present study, we have analysed the contribution of these proposed SIMs in RNF4 in the recognition of poly-SUMO chains and their proteasomal targeting by RNF4 in cells. The interaction of RNF4 with mono-SUMOs and poly-SUMOs was analysed by ITC (isothermal titration calorimetry). We show that these data correlate to the disruption of PML-NBs, which depends on the activity of RNF4 to degrade PML, and the proteasomal targeting of model substrate proteins in *Saccharomyces cerevisiae*.

## EXPERIMENTAL

### Constructs and protein expression

For expression in *Escherichia coli*, linear poly-SUMO chains were generated as described previously [24] with slight modifications. After the combined PCR/ligation reaction, the 4×SUMO2-ΔN11 or 4×SUMO1-ΔN15 DNA polymers were separated from smaller polymers by agarose gel electrophoresis and individually extracted (Macherey–Nagel). These were then subjected to a second PCR with primers to create BamHI and XhoI restriction sites, a C-terminal GG motif and a stop codon. The resulting fragments coding for di-, tri- and tetra-SUMO2-ΔN11 or SUMO1-ΔN15 chains were amplified and cloned into pGEX-4T2. Expression from these plasmids resulted in GST-tagged artificial SUMO2-ΔN11 or SUMO1-ΔN15 monomers or linear-linked chains with up to four monomers. Each monomer in these chains is separated by RS replacing the natural GG motif. A similar strategy was used to design C-terminally GFP–HA_2_-tagged linear poly-SUMOs in the copper-inducible Ycplac22 vector for expression in *S. cerevisiae*. The N-terminal SUMO2 moiety in these linear SUMO2 fusion proteins contained the authentic N-terminus extended by a FLAG tag, whereas the first 11 residues were missing in the subsequent SUMO2 moieties within a chain in order to mimic the authentic distance between SUMOs in a Lys^11^-linked chain. The C-terminal glycine residue of each SUMO2 moiety was mutated to a leucine residue to prevent cleavage by SUMO-deconjugating enzymes. RNF4 was cloned as described previously [22]. For ITC measurements, the N-terminal 1–125 amino acids were subcloned into pGEX-4T2 (GE Healthcare) by PCR using BamHI/EcoRI restriction sites. Mutations of SIMs were introduced by site-directed mutagenensis with pairs of complementary primers. Thus sequences within SIM1 (IELV), SIM2 (VDLT), SIM3 (VDLT) or SIM4 (VVIV) were replaced by AAAA. For expression in *S. cerevisiae*, these constructs were fused to a sequence encoding an N-terminal FLAG-tag and expressed from the galactose-inducible *GAL1* promoter in a low copy (CEN/URA3) vector.

The mono-SUMOs and linear poly-SUMO1 and poly-SUMO2 proteins as well as all RNF4 constructs were expressed as GST-fusion proteins from *E. coli* Rosetta™ 2(DE3)pLysS cells (Novagen) cultivated in LB medium with 100 μg/ml ampicillin and 30 μg/ml chloramphenicol after induction with 0.1 mM IPTG at 20°C for 12 h. Cells were lysed by sonication in GST-buffer I (50 mM Tris/HCl, pH 7.5, 150 mM NaCl and 2 mM DTT) in the presence of protease inhibitors (1 μg/ml aprotinin, 1 μg/ml leupeptin, 1 μg/ml pepstatin, 200 μM pefabloc and 100 μM PMSF). For tetra-SUMOs and RNF4 variants, 1.5% sarkosyl, 0.1% Triton X-100 and 5% glycerol were added. The proteins were affinity purified using glutathione beads (Protino® Glutathione Agarose 4B, Macherey–Nagel) and eluted by thrombin cleavage. The supernatant was subjected to anion exchange chromatography and further purified by SEC (size-exclusion chromatography) into ITC buffer (50 mM Tris/HCl, pH 7.5, 150 mM NaCl, 2 mM DTT and 5% glycerol) or in crystallization buffer (20 mM Tris/HCl, pH 7.5, and 100 mM NaCl). Native SUMO2 chains were produced by *in vitro* SUMOylation as described recently [38] and subsequently purified by affinity chromatography and SEC.

### Proteasomal targeting of linear SUMO chains tagged with GFP in yeast

wt (wild-type) *S. cerevisiae* strains (JD47-13C) containing centromeric (low copy) plasmids expressing various (SUMO2)*n*–GFP–HA_2_ (*n*=24) fusions were grown overnight in minimal medium composed of 0.67% yeast nitrogen base with 2% raffinose as a carbon source. Cells from the overnight cultures were used to inoculate cultures with minimal media containing 2% galactose at a starting attenuance measured at 600 nm of 0.25. After a 2.5 h incubation period at 30°C, 100 μM CuSO_4_ was added to induce expression of the SUMO–GFP constructs. The cells were grown until they reached *D*_600_ ~0.8 in the exponential phase. Extracts were then prepared by boiling the cells in buffer containing 62.5 mM Tris/HCl, pH 6.8, 2% SDS, 10% glycerol, 8 M urea, m-Cresol Purple and 1% 2-mercaptoethanol. The levels of substrates and, as a loading control, levels of CDC11 were analysed by Western blotting and detection with an Odyssey infrared fluorescence imaging system (LI-COR).

### Isothermal titration calorimetry

Binding of peptides and proteins to SUMOs was investigated by ITC using either a VP-ITC or an ITC_200_ (GE Healthcare). All experiments were performed in ITC buffer at 25°C. The peptides or proteins were injected in 20–30 steps up to a 2–3-fold molar excess. Titration curves were fitted to the data using ORIGIN (supplied by the manufacturer). The values were averaged from two to six titrations. Protein concentrations were determined by measuring the absorption at 280 nm. Peptides were purchased (PANATecs) or synthesized in-house and weighed on an analytical balance.

### Immunofluorescence and antibodies

HeLa B-cells were grown on coverslips and fixed in PBS with 3% paraformaldehyde. After permeabilization in PBS with 0.2% saponin (Sigma–Aldrich), cells were blocked in PBS, 3% BSA and 0.2% saponin. Primary and Alexa Fluor®-labelled secondary antibodies (Invitrogen) were applied in blocking buffer. Coverslips were embedded in ProLong® Gold Antifade (Invitrogen) and examined using a Zeiss Axioplan2 fluorescence microscope. Proteins and epitope tags were detected with the following antibodies against PML (A167 and A168; Bethyl), HA (haemagglutinin) (3F10; Roche, 16B12; Covance) and CDC11 (SC-7170; Santa Cruz Biotechnology). The secondary antibodies were labelled with Alexa Fluor® 546, Alexa Fluor® 647 or Alexa Fluor® 670 (Invitrogen), or IRDye800 (Rockland). For quantification of PML-NBs, cells were acquired using a Zeiss Meta 510 Confocal microscope, 63× objective NA (numerical aperture) 1.4 and appropriate laser and filter settings. Z-stacks were taken according to Nyquist criteria. Microscopic pictures were analysed with Volocity 6.2 Cellular Imaging & Analysis Software (PerkinElmer). Transfected cells were automatically identified within the 546 nm channel and the amount of PML-NBs (647 nm channel) that were localized within the nuclei of transfected cells were automatically counted.

## RESULTS AND DISCUSSION

### The interaction of RNF4 and poly-SUMO chains is driven by avidity

In order to analyse the contribution of the four putative SIMs in RNF4 (Figure 1A) to the binding of SUMOylated substrates, we tested the interaction of full-length RNF4 and of a construct comprising the entire N-terminal part of RNF4 upstream of the RING domain (subsequently referred to as ‘SIM domain’) with mono-SUMO1 and mono-SUMO2 and with linear SUMO–SUMO fusion proteins of variable length [24]. These SUMO fusion proteins lacked the N-terminal 11 residues of each SUMO, thus retaining the natural distance as it occurs within a natural Lys^11^-linked SUMO chain.

ITC measurements revealed that the affinity of the RNF4-SIM domain for mono-SUMOs is very weak (Figures 1B and 1C) although the core sequences of SIM2, SIM3 and SIM4 match well with the consensus sequences, whereas the proposed SIM1 has only remote similarity [9]. The observed low-affinity points to the importance of flanking acidic residues for a high-affinity SUMO–SIM interaction, which are less abundant in the SIMs of RNF4 when compared with the SIMs of the tightly SUMO-binding proteins PIAS [protein inhibitor of activated STAT (signal transducer and activator of transcription)] [10,11], MCAF-1 (MBD1-containing chromatin-associated factor 1) [13] and Daxx [14,15]. The interaction of the SIM domain with linear di-SUMOs was stronger, with similar dissociation constants (*K*_D_) of 17 μM for di-SUMO1 and 14 μM for di-SUMO2. These data indicate the importance of the multivalency of the interaction, which is apparently not driven by a single high-affinity interaction. Importantly, we found almost no difference in binding of the linear di-SUMO fusions compared with the binding affinity of ‘native’ Lys^11^-linked di-SUMO2 chains (*K*_D_=11 μM) (Figure 1D). This validates that linear SUMO fusions are a suitable model for poly-SUMOs and that the SIM domain of RNF4 recognizes the SUMO chain only via the established SUMO–SIM interaction and not by additional binding to the branching point at Lys^11^. This is in contrast with ubiquitin, where conjugates linked linearly or via different lysine residues are specifically recognized due to their relative orientation in the chain and by direct contacts to the linking residues [5,40]. Extension of the SUMO chains from di-SUMO to tri-SUMO did not result in an increase in affinity of the SIM domain (*K*_D_=19 μM). Elongation of the SUMO chain to four moieties, however, resulted in a significant increase in affinity with a *K*_D_ value of 4.3 μM for tetra-SUMO1 and 1.6 μM for tetra-SUMO2. These results indicate that longer SUMO chains interact with more than two SIMs and suggest that the distance between SUMO units in a chain is of relevance. Binding of full-length dimerized RNF4 to SUMO chains was stronger as compared with the monomeric SIM domain, especially for short SUMO chains. Furthermore, the crystal structure of linear di-SUMO2 indicates that SUMO chains are very flexible (Supplementary Figure S1 and Supplementary Table S1 at http://www.biochemj.org/bj/457/bj4570207add.htm) as was proposed earlier [41]. The last residue at the C-terminus of the first SUMO monomer in the proposed di-SUMO2 and the first N-terminal residue of the second SUMO monomer that are resolved in the structure are 14.1 Å (1 Å=0.1 nm) apart. The linker region in between comprises 11 residues and is not resolved, indicating that it is structurally flexible. The residues of this stretch can easily cover the distance of 14.1 Å in an extended form. Therefore a SUMO chain in solution is expected to be able to adopt multiple conformations to interact with the SIMs in unstructured regions of SUMO-binding proteins.

**Figure 2:**
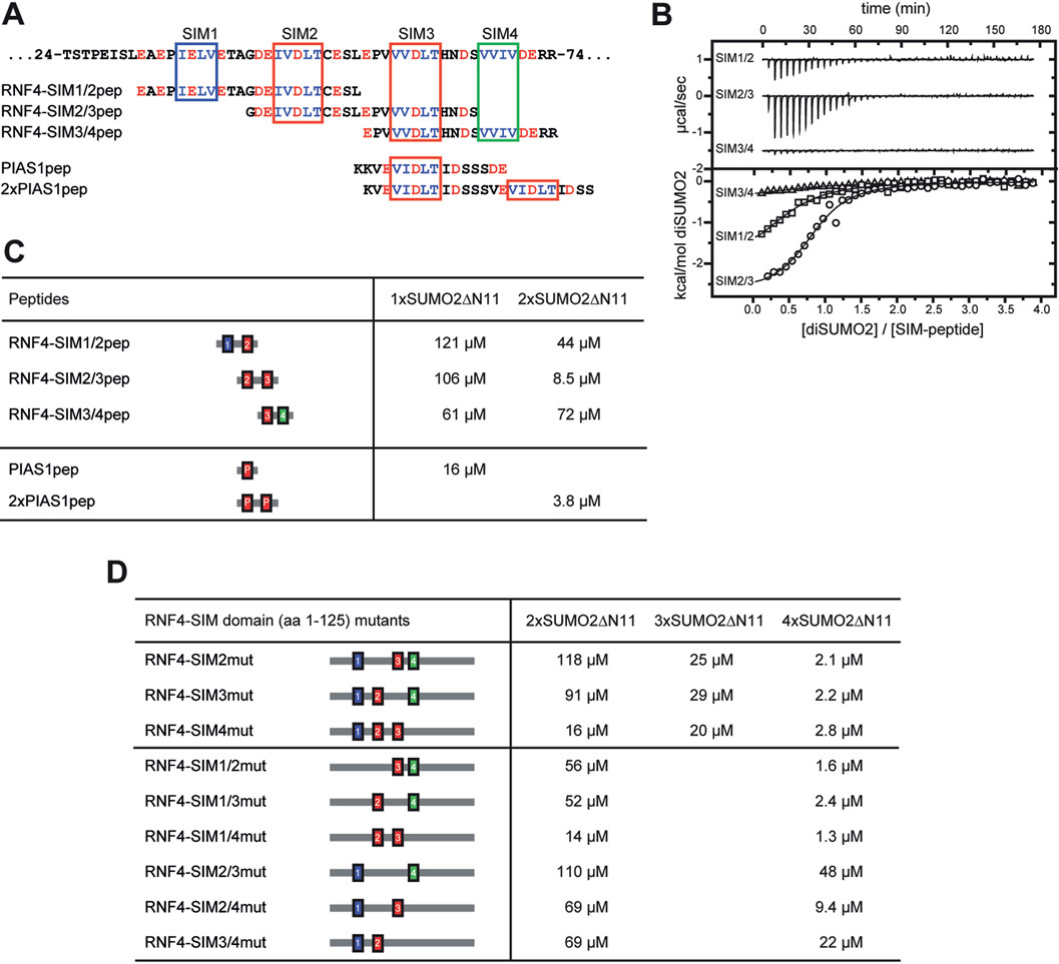
Calorimetric titration of RNF4 peptides and SIM mutants with di-SUMO2 and tetra-SUMO2 (**A**) Primary sequence of the SUMO-binding region of RNF4 (top) and of the RNF4- and PIAS-derived SIM peptides (bottom). Colour coding is as in Figure 1. (**B**) Profiles of typical calorimetric titrations of di-SUMO2 into a solution of RNF4-SIM1/2 peptide, RNF4-SIM2/3 peptide or RNF4-SIM3/4 peptide (upper panel, top to bottom) and the integrated data, normalized to the concentration of di-SUMO2 (lower panel). (**C** and **D**) Dissociation constants of the interaction of polymeric chains of SUMO2 with RNF4 and PIAS-derived peptides and with SIM mutants of the RNF4-SIM domain.

In order to dissect the contributions of the SIMs, we designed peptides that each comprise a tandem of two SIMs from RNF4 and titrated them with a linear di-SUMO2 fusion (Figures 2A–2C). All three peptides interacted only weakly with mono-SUMO2. When titrated with linear di-SUMO2, the SIM2/3 peptide comprising the two type b SIMs displayed the lowest *K*_D_ (8.5 μM) of the three peptides. Interestingly, this *K*_D_ is very similar to the one of the whole RNF4-SIM domain for di-SUMO2. Surprisingly, the SIM3/4 peptide showed a similarly low affinity for di-SUMO2 as compared with mono-SUMO2. This may be due to the spacing between SIM3 and SIM4, which is shorter than the one between SIM2 and SIM3, thereby preventing a simultaneous interaction of both SIMs with di-SUMO2. To analyse further the avidity effect of clustered SIMs, we compared this with the binding of a single PIAS1 peptide to SUMO2 and of a synthetic tandem PIAS1 SIM peptide to di-SUMO2. PIAS displays one of the tightest SUMO interactions described in the literature [10,11] and the VIDLT core of its SIMb, as well as the C-terminally flanking regions, are optimized for a high affinity for mono-SUMO [9,38]. Therefore the affinity of the single PIAS1 peptide for mono-SUMO2 (*K*_D_=16 μM) was much stronger than the binding of any RNF4-derived tandem SIM peptide. The combination of two such sequences in the tandem PIAS1 peptide yielded a high affinity for di-SUMO2 (*K*_D_=3.8 μM), which exceeded the affinities of the RNF4-SIM2/3 peptide.

### Analysis of individual SIMs in RNF4

To analyse the recognition of SUMO chains in more detail, we created mutations of one or several of the SIMs in the RNF4-SIM domain (Figure 2D). Titrations of single SIM mutants with linear di-SUMO2 and tetra-SUMO2 confirmed that SIM2 and SIM3 contribute most to the binding of SUMO chains. The SIM4 mutant displayed a 4–5-fold stronger binding to diSIMO2 than the SIM2 and SIM3 mutants, but its interaction with tetra-SUMO2 was only slightly weaker compared with the two other single mutants. The affinities of SIM double mutants retaining two adjacent SIMs (SIM1/2mut, SIM1/4mut and SIM3/4mut) for di-SUMO2 resembled those of the corresponding tandem peptides (SIM3/4pep, SIM2/3pep and SIM1/2pep). Not surprisingly, the SIM2/3 double mutant displayed the weakest affinity for di-SUMO2. All double mutants containing a mutation of SIM1 and another SIM bound with similar affinity to di-SUMO2 and tetra-SUMO2 as the corresponding single mutants with a wt SIM1. These results are in line with previous data that SIM1 does not contribute much, if at all, to binding [24]. Compared with the wt RNF4-SIM domain, the single SIM mutants displayed only slightly weaker binding to tetra-SUMO2 with *K*_D_ values in the low micromolar range. Despite that, several of these mutants have been shown previously to be highly impaired with respect to binding and ubiquitylation of SUMO chains *in vitro* [24]. The double mutants combining mutations of SIM2, SIM3 or SIM4 displayed 5–20-fold weaker binding of tetra-SUMO2 as compared with the wt.

**Figure 3:**
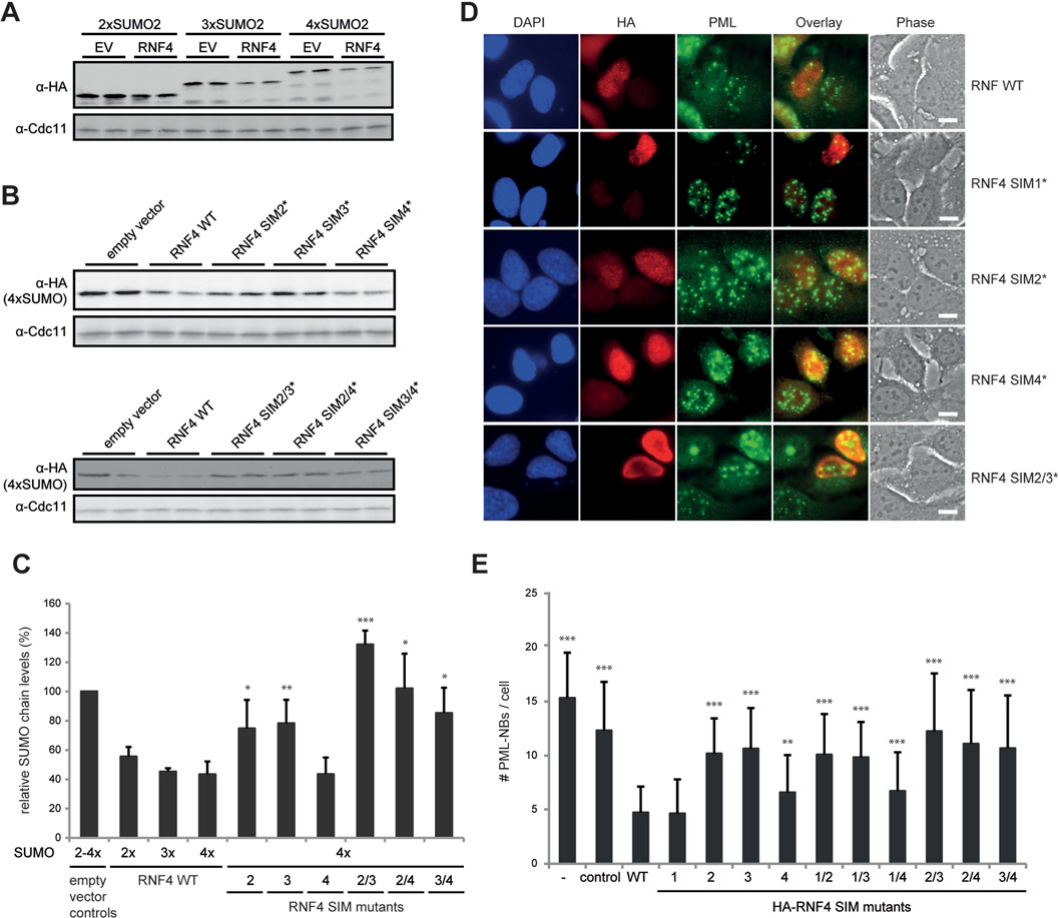
Analysis of RNF4 activity in cell-based assays (**A**) Proteolytic targeting of poly-SUMO chains tagged with GFP–HA_2_ by RNF4 in yeast. Western blot extracts from *S. cerevisiae* cells overexpressing wt FLAG-tagged RNF4 and di-, tri- and tetra-SUMO2 chains C-terminally fused to GFP–HA_2_ (in duplicates). (**B**) Western blot extracts from *S. cerevisiae* cells overexpressing mutant FLAG-tagged RNF4 and tetra-SUMO2–GFP–HA_2_ (in duplicates). (**C**) Quantification of experiments as shown in (**A**) and (**B**) obtained by normalization to the internal loading control CDC11. The levels of the respective poly-SUMOs in the absence of RNF4 were set to 100%. (**D**) Disruption of PML-NBs by overexpression of RNF4 in HeLa cells. Immunofluorescence of HeLa cells transfected with wt or mutant HA-tagged RNF4 stained for DNA (DAPI), transfected RNF4 (HA) and endogenous PML. FLAG-tagged GFP was used as transfection control. (**E**) Quantification of experiments as shown in (**D**) and in Supplementary Figure S4 at http://www.biochemj.org/bj/457/bj4570207add.htm. Between 30 and 60 cells were analysed for each condition. Significance levels in (**C**) and (**E**) are as follows: *P≤0.05, **P≤0.01 and ***P≤0.001, in relation to wt RNF4.

To correlate the strength of the interaction of RNF4 and its SIM mutants with poly-SUMOs to the activity of RNF4 in living cells, we made use of our previous findings that RNF4 is functional in yeast and disrupts PML-NBs in mammalian cells [17,22]. We therefore co-expressed combinations of full-length wt RNF4 or its SIM mutants with poly-SUMO chains that retained the native N-terminus on the first SUMO moiety and were C-terminally tagged with GFP–HA in *S. cerevisiae*. As shown in Figures 3(A)–3(C), the poly-SUMO–GFP–HA reporter constructs were efficiently targeted for degradation. Compared with the control transformants not expressing RNF4, the levels of di-SUMO2 were reduced to approximately 55%, and this effect was enhanced if the chain was composed of three or four SUMOs (45% and 43% of control levels respectively). Next, we tested the activity of the SIM mutants to target the tetra-SUMO–GFP–HA construct for degradation. In these experiments, the levels of tetra-SUMO–GFP–HA were only slightly reduced by coexpression with the RNF4 carrying single mutations inactivating either SIM2 or SIM3, whereas the SIM4 mutant was as active as the wt. SIM2/4 and SIM3/4 double mutants were also highly impaired in targeting tetra-SUMO2. In case of the SIM2/3 mutant, the levels of tetra-SUMO2 were even higher than in control cells (Figures 3B and 3C). This could be explained by protection of tetra-SUMO2 from ubiquitylation by endogenous STUbLs in *S. cerevisiae* by competition of the mutant RNF4 with these proteins for the ubiquitin-conjugating enzymes Ubc4 and Ubc5. Importantly, levels of the mutant RNF4 proteins were at least as high as those of wt RNF4 supporting the notion that their reduced *in vivo* function is indeed due to impairment of binding (Supplementary Figure S2 at http://www.biochemj.org/bj/457/bj4570207add.htm). To verify that the differences in substrate steady-state levels observed following coexpression of RNF4 or its mutant variants are indeed due to differences in protein turnover and not to differences in expression, we performed pulse–chase experiments. Specifically, we compared the stability of the linear tetra-SUMO2 reporter construct in the absence or presence of RNF4, or its SIM2 mutant version (Supplementary Figure S3 at http://www.biochemj.org/bj/457/bj4570207add.htm). The results show that the reporter protein is fairly stable over the chase period in the absence of RNF4, whereas it is degraded upon coexpression of RNF4. Consistent with the steady-state data, the turnover kinetics of the reporter protein are significantly lower when the mutant RNF4-SIM2 is coexpressed instead of its wt counterpart. These data therefore confirm that the difference in steady-state levels detected in Figure 3 reflect differences in turnover rates resulting from RNF4 function.

Next, we analysed the impact of transfected wt or mutant RNF4 on PML-NBs in HeLa cells by immunofluorescence microscopy (Figure 3D, and Supplementary Figure S4 at http://www.biochemj.org/bj/457/bj4570207add.htm). The results of PML-NB quantification using computer-based automatic image analysis are shown in Figure 3(E). Comparable results were obtained by manual double-blind counting (results not shown). In the absence of interferon stimulation, HeLa cells contain on average 15 PML-NBs. Transfection with a GFP control plasmid resulted in a slight reduction to 12 PML-NBs. This number was reduced to approximately five PML-NBs by transfection of wt RNF4 or the SIM1 mutant. Mutation of the other single SIMs in RNF4 impaired the disruption of PML-NBs (ten PML-NBs for the SIM2 mutant, 11 for the SIM3 mutant and seven for the SIM4 mutant). Consistent with the ITC results, a mutation of SIM1 together with either SIM2, SIM3 or SIM4 showed no additional effect compared with the single mutants confirming that SIM1 contributes little, if at all, to the activity of the protein. Even stronger effects were observed when SIM2/3, SIM2/4 or SIM3/4 were mutated, which completely abrogated the activity of RNF4 to disrupt PML-NBs. Overall, RNF4 mutants showed remarkably similar relative activities in the two *in vivo* assays using either yeast or HeLa cells. Since these activities correlate with the binding affinities for di-SUMO2, but not for tetra-SUMO2 (compare Figures 2D, 3C and 3E), and since wt RNF4 efficiently targets di-SUMO2 in yeast, we conclude that proteins modified with a chain of two SUMO moieties are targeted by RNF4. The discrepancy between these results and an earlier report showing that chains of more than two SUMO moieties were necessary for efficient ubiquitylation by RNF4 *in vitro* [24] can be resolved by considering the protein concentrations. In those *in vitro* assays they were below the dissociation constants and therefore only a small proportion of RNF4 interacted with short SUMO chains under these conditions.

### Concluding remarks

**Figure 4:**
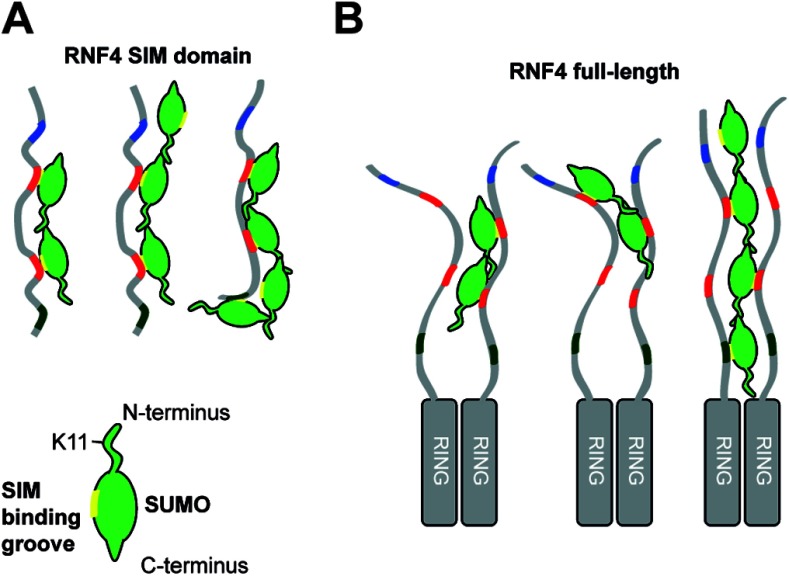
Model for the recognition of SUMO chains by RNF4 (**A**) SIM2 and SIM3 are necessary and sufficient for an interaction of the RNF4-SIM domain with a chain of two SUMO2 proteins (left). The sequence of SIM1 and the spacing between SIM3 and SIM4 prevent a stronger interaction with chains of three SUMOs (middle). Owing to the flexibility of both the SUMO chain and the SIM domain, longer chains can interact with a higher affinity (right). (**B**) In the case of full-length RNF4, the dimerization of the RING domain leads to a higher avidity of RNF4 for SUMO chains due to the interaction with SIMs from both monomers. This flexibility and the relatively low-binding affinities of the individual SIMs leads to a dynamic equilibrium between several binding conformations of RNF4 and SUMO chains, which bring the last SUMO in the chain into the proximity of the E2 to allow transfer of a ubiquitin to Lys^11^ in SUMO and the subsequent elongation of the ubiquitin chain.

The degradation of poly-SUMOylated PML-RARα by RNF4 forms the basis for the therapy of APL in response to arsenic trioxide [22–24,26,42]. Our work, which is the first quantitative study on the interaction of a protein with multiple SIMs with a poly-SUMO chain, shows how the sequence and spacing of the SIMs in the N-terminal domain of RNF4 determines its substrate specificity (Figure 4). We conclude that the tandem of SIM2 and SIM3 is necessary and sufficient for an interaction of the SIM domain with chains of at least two SUMOs. SIM4 can only contribute to binding if the chain is elongated to at least four SUMOs since the spacing between SIM3 and SIM4 is too close for an interaction with two adjacent SUMOs. SIM1 is not contributing much, if anything, to the binding of SUMO chains, and therefore RNF4 has only three functional SIMs. For endogenous RNF4 and PML this leads to a finely tuned balanced situation. The combination of a low concentration of RNF4 and of short SUMO chains on PML results in a dynamic recruitment of RNF4 to PML-NBs, but not to their disruption. External stimuli, such as DNA damage or arsenic treatment, result in the formation of more and longer SUMO chains. The multiple low-affinity SUMO–SIM interactions enable RNF4 to specifically recognize SUMO chains with a high avidity leading to ubiquitylation of its substrates. Theoretically it is possible that multiple mono-SUMOylations on distinct lysine residues of a protein can also trigger ubiquitinylation by RNF4 if the SUMOs are in close proximity [43]. The importance of the strength of individual SIMs, as well as of their location and spacing, demonstrated in the present study suggests that other SUMO-binding proteins and STUbLs will display distinct binding specificities due to differences in these parameters.

## Online data

Supplementary data
