# Supplementary material for: Multivalent interactions of the SUMO-interaction motifs in RING finger protein 4 determine the specificity for chains of the SUMO
Source: Biochem J. 2013 Dec 10;457(Pt 1):207–14. doi: 10.1042/BJ20130753 (PMC3901395; doi:10.1042/BJ20130753)
Supplement: Supplementary data [file bj4570207add.pdf]

BJ Signal

Biochem. J. (2014) **457**, 207–214 (Printed in Great Britain) doi:10.1042/BJ20130753

online data

SUPPLEMENTARY ONLINE DATA

Multivalent interactions of the SUMO-interaction motifs in RING finger protein 4 determine the specificity for chains of the SUMO

Kirstin KEUSEKOTTEN*†[1], Veronika N. BADE*†[2], Katrin MEYER-TESCHENDORF*†[2], Annie Miriam SRIRAMACHANDRAN†[2], Katrin FISCHER-SCHRADER‡, Anke KRAUSE*†, Christiane HORST*†, Günter SCHWARZ‡*, Kay HOFMANN†, R. Jürgen DOHMEN† and Gerrit J. K. PRAEFCKE*†[3]

*Center for Molecular Medicine Cologne (CMMC), University of Cologne, 50674 Köln, Germany
†Institute for Genetics, University of Cologne, 50674 Köln, Germany
‡Institute for Biochemistry, University of Cologne, 50674 Köln, Germany

EXPERIMENTAL

Crystallization and structure determination of linear di-SUMO2-ΔN11

Concentrated di-SUMO2-ΔN11 (10 mg/ml) in crystallization buffer was used for crystallization experiments by hanging-drop vapour diffusion. Crystals grew in a solution of 0.1 M Tris/HCl, pH 8.0, 28 % PEG 350 MME (Qiagen) and 0.05 % dioxane within 2–4 days at room temperature (21 °C). Obtained crystals were flash-frozen from the mother liquor, and an X-ray diffraction dataset was collected at 100 K with a Supernova diffractrometer from Agilent Technologies. The data were indexed, integrated and scaled with XDS [1]. Molecular replacement was carried out with PHASER [2] using PDB code 1WM3 as a search model and subsequent refinement with Refmac 5 [3]. Manual corrections were performed with COOT [4]. Refinement statistics are listed in Table S1. The final atomic co-ordinates, together with experimental structure factors, were deposited at the PDB under accession code 4BKG.

Experiments in yeast

To monitor and compare the cellular levels of FLAG-tagged version of wt and its SIM mutant variants, the extracts were analysed by anti-FLAG antibody (M5 antibody; Sigma) using Western blot analysis and ECL detection (Figure S2). To follow proteolytic turnover of FLAG–(SUMO2)4–GFP–HA_2, we performed pulse–chase analyses as described previously [5], using 2 min pulse labelling with [^{35}S]methionine, anti-HA antibody immunoprecipitation (EZview™ Red Anti-HA Affinity Gel, Sigma–Aldrich) and phosphorimaging (Typhoon Trio Imager; GE Healthcare) (Figure S3).

[1] Present address: MRC Laboratory of Molecular Biology, PNAC Division, Cambridge Biomedical Campus, Francis Crick Avenue, CB2 0QH, U.K.
[2] These authors contributed equally to this work.
[3] To whom correspondence should be addressed (email: gpraefck@uni-koeln.de).
The structural co-ordinates reported will appear in the PDB under code 4BKG.

© 2014 The Author(s)
The author(s) has paid for this article to be freely available under the terms of the Creative Commons Attribution Licence (CC-BY) (http://creativecommons.org/licenses/by/3.0/) which permits unrestricted use, distribution and reproduction in any medium, provided the original work is properly cited.

Biochemical Journal www.biochemj.org

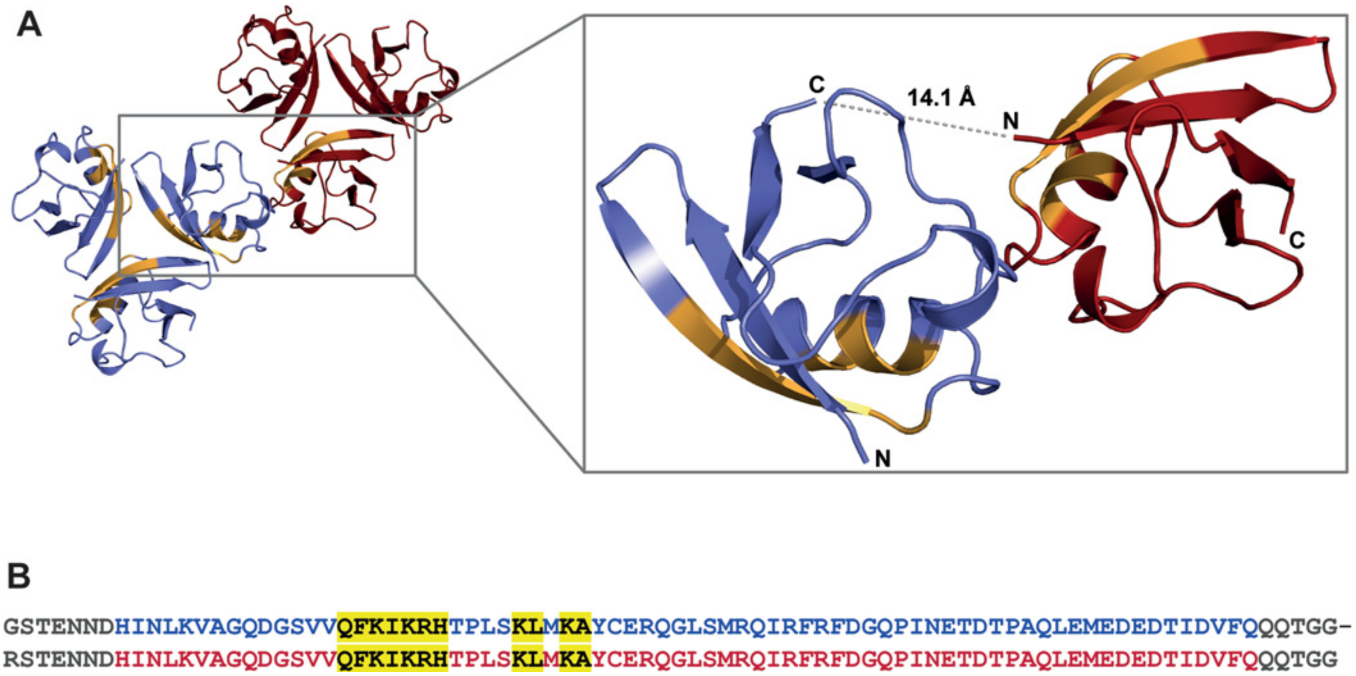

Figure S1 Structure of linear di-SUMO2-ΔN11

(**A**) Crystal structure of linear di-SUMO2. Starting with one SUMO2 monomer in the asymmetric unit, the crystallographic oligomerization state resembles that of mono-SUMO2 (e.g. PDB code 1WM3 [6]), and is shown in the present study as two trimers of SUMO2. As degradation of linear di-SUMO2 could be excluded by SDS/PAGE and Western blotting of purified crystals, the dimer of SUMO2 is formed via crystallographic symmetry axes. Two monomers (boxed and enlarged) are oriented with their termini towards each other so that the gap of 14.1 Å can be covered easily by the missing 11 residues connecting the SUMO monomers in the amino acid sequence. (**B**) Sequence of crystallized di-SUMO2. Blue and red residues indicate the SUMO monomers respectively. SIM-binding residues are coloured yellow. Grey residues are not resolved in the crystal structure.

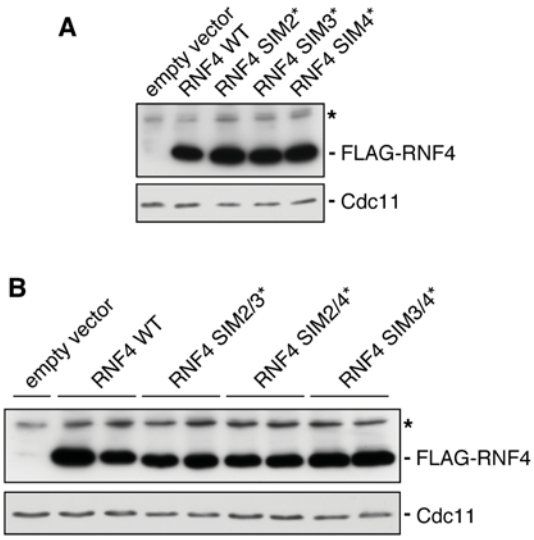

Figure S2 Comparison of wt and mutant RNF4 levels in yeast transformants

(**A**) Extracts from yeast cells expressing wt or its single SIM mutant versions were analysed by SDS/PAGE and anti-FLAG antibody Western blotting. (**B**) Same as in (**A**), but with extracts from cells expressing wt or its SIM double mutant versions. Asterisks indicate a protein that cross-reacted with the antibody. The blots were re-probed with anti-Cdc11 antibody to control for differences in the amounts of loaded protein.

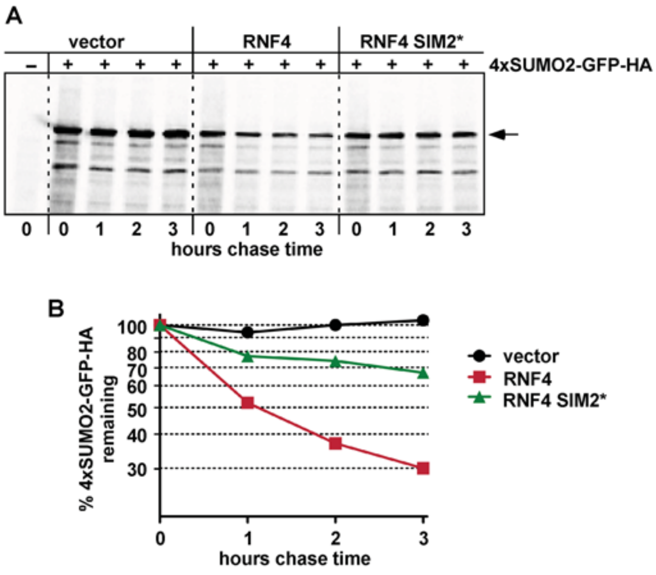

Figure S3 Pulse–chase analysis of (SUMO2)4–GFP–HA$_2$ turnover

(**A**) Phosphorimager detection of anti-HA antibody immunoprecipitated substrate protein extracted from the cells after the indicated chase times. Apart from a plasmid expressing (SUMO2)4–GFP–HA$_2$, the cells were transformed with either an empty *URA3*-marked vector or plasmids expressing either wt RNF4 or its SIM2 mutant version. A transformant not expressing (SUMO2)4–GFP–HA$_2$ was used as control to confirm that the observed signals are specific for the HA-tagged substrate. (**B**) Quantification of the (SUMO2)4–GFP–HA$_2$ signals in (**A**) (band indicated by an arrow).

© 2014 The Author(s)
The author(s) has paid for this article to be freely available under the terms of the Creative Commons Attribution Licence (CC-BY) (http://creativecommons.org/licenses/by/3.0/) which permits unrestricted use, distribution and reproduction in any medium, provided the original work is properly cited.

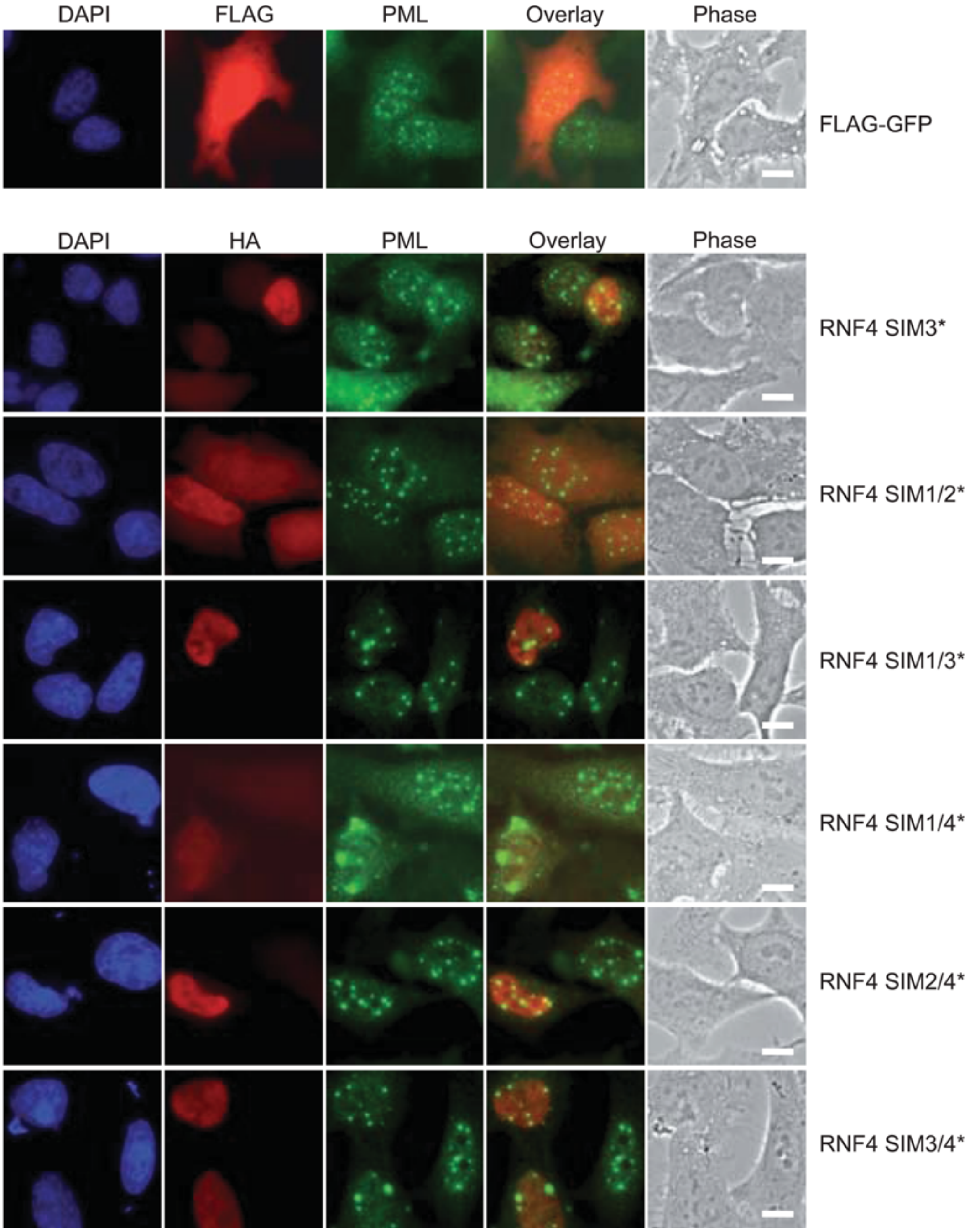

Figure S4 Analysis of RNF4 SIM mutants in HeLa cells

Disruption of PML-NBs by overexpression of RNF4 in HeLa cells. Immunofluorescence of HeLa cells transfected with FLAG–GFP or mutant HA-tagged RNF4 stained for DNA (DAPI), transfected RNF4 (HA) and endogenous PML.

© 2014 The Author(s)
The author(s) has paid for this article to be freely available under the terms of the Creative Commons Attribution Licence (CC-BY) (http://creativecommons.org/licenses/by/3.0/) which permits unrestricted use, distribution and reproduction in any medium, provided the original work is properly cited.

Table S1 Data collection and refinement statistics

Values in parentheses refer to the highest resolution shell.

Parameter	c2p-2
Data collection	
Wavelength (Å)	1.54
Space group	*H*3
Unit cell	
a, *b*, *c* (Å)	74.99, 74.99, 33.27
α, β, γ (°)	90, 90, 120
Resolution (Å)	2.1
Redundancy	2.8 (1.4)
Completeness (%)	94.8 (73.9)
$I/\sigma I$	12.5 (1.9)
R_{meas} (%)	8.4 (57.2)
Refinement statistics	
Resolution range (Å)	23.2-2.1
Number of unique reflections	3842
R_{work} (%)	17.9
R_{free} (%)*	23.1
Number of residues	73 (His^{17}–Gln^{89})
Number of water molecules	27
Average *B*-factor ($Å^2$)	28.2
RMSDs	
Bond length (Å)	0.019
Bond angles (°)	1.88
RAMACHANDRAN analysis	
Favoured (%)	98.6
Allowed (%)	1.4

**R*-factor for the randomly selected 5 % of the data, which is not used for refinement.

REFERENCES

1 Kabsch, W. (2010) Xds. Acta Crystallogr., Sect. D: Biol. Crystallogr. **66**, 125–132
2 McCoy, A. J. (2007) Solving structures of protein complexes by molecular replacement with Phaser. Acta Crystallogr., Sect. D: Biol. Crystallogr. **63**, 32–41
3 Murshudov, G. N., Vagin, A. A. and Dodson, E. J. (1997) Refinement of macromolecular structures by the maximum-likelihood method. Acta Crystallogr., Sect. D: Biol. Crystallogr. **53**, 240–255
4 Emsley, P. and Cowtan, K. (2004) Coot: model-building tools for molecular graphics. Acta Crystallogr., Sect. D: Biol. Crystallogr. **60**, 2126–2132
5 Ramos, P. C., Höckendorff, J., Johnson, E., Varshavsky, A. and Dohmen, R. J. (1998) Ump1p is required for maturation of the 20S proteasome, and becomes its substrate upon completion of the assembly. Cell **20**, 489–499
6 Huang, W. C., Ko, T. P., Li, S. S. and Wang, A. H. (2004) Crystal structures of the human SUMO-2 protein at 1.6 A and 1.2 A resolution: implication on the functional differences of SUMO proteins. Eur. J. Biochem. **271**, 4114–4122

Received 12 June 2013/18 October 2013; accepted 23 October 2013
Published as BJ Immediate Publication 23 October 2013, doi:10.1042/BJ20130753

© 2014 The Author(s)
The author(s) has paid for this article to be freely available under the terms of the Creative Commons Attribution Licence (CC-BY) (http://creativecommons.org/licenses/by/3.0/) which permits unrestricted use, distribution and reproduction in any medium, provided the original work is properly cited.
